# Behavioral correlates of cheating: Environmental specificity and reward expectation

**DOI:** 10.1371/journal.pone.0186054

**Published:** 2017-10-26

**Authors:** Michael Isakov, Arnav Tripathy

**Affiliations:** 1 Lincoln-Sudbury Regional High School, Sudbury, Massachusetts, United States of America; 2 Department of Mathematics, Stanford University, Stanford, California, United States of America; University of the Basque Country, SPAIN

## Abstract

Academic dishonesty has been and continues to be a major problem in America’s schools and universities. Such dishonesty is especially important in high schools, where grades earned directly impact the academic careers of students for many years to come. The rising pressure to get the best grades in school, get into the best college, and land the best paying job is a cycle that has made academic dishonesty increase exponentially. Thus, finding the widespread roots of cheating is more important now than ever. In this study, we focus on how societal norms and interactions with peers influence lying about scores in order to obtain a benefit in a high school population. We show that (1) the societal norms that go hand in hand with test-taking in school, as administered by a teacher, significantly dampen small-scale dishonesty, perhaps suggesting that context-dependent rewards offset cheating; (2) providing reminders of societal norms via pre-reported average scores leads to more truthful self-reporting of honesty; (3) the matrix search task was shown to not depend on class difficulty, confirming its effectiveness as an appropriate method for this study; (4) males seem to cheat more than females; and (5) teenagers are more dishonest earlier in the day. We suggest that students understand that cheating is wrong, an idea backed up by the literature, and that an environment which clearly does not condone dishonesty helps dampen widespread cheating in certain instances. This dampening effect seems to be dependent on the reward that students thought they would get for exaggerating their performance.

## Introduction

Cheating is a complex and often contradictory phenomenon. It is well-established that humans choose cooperative options in many realistic scenarios, with cooperation or “playing fair” often shaped by social heuristics [1–3]. However, cheating in specific situations is still seen to be highly prevalent [4]. One particularly intriguing phenomenon is academic dishonesty. This is perhaps most evident in high schools, where grades earned are thought to pave the path to students’ futures. The rapidly rising expectations of high schoolers has brought levels of cheating to dizzying heights; as many as 90 percent of students reported cheating in high school or in college [5]. This is up from just under 25 percent in the 1940s. Maturity is often thought to be one of the main constraining factors for cheating. This is true in that younger students (freshman and sophomores in high school), cheat more than older kids on tests, homework, and essays [6, 7]. However, this trend is ‘reset’ upon entering a new environment, that is, younger students in college are more likely to cheat than older students in high schools, with overall cheating being similar in different environments. Thus, it appears that there are other things impacting dishonesty apart from physiology, knowledge, and general maturity.

Although cheating has important applications for school related activities, individuals who engage in academic dishonesty are also more likely to engage in unethical behavior outside of school [8, 9]. This is because individuals who succumb to the temptation to cheat have been shown to have less self-control than those who do not, a key point with many societal ramifications [5]. Considering that dishonesty is so common in academic and professional environments, we expect to find widespread dishonesty and corruption in other areas. With that in mind, it must be noted that young children do not have nearly the same ability or willingness to be dishonest, suggesting that it is not an innate property of humans.

Unethical practices by corporations costs the US tens of billions of dollars each year. However, things that ordinary people would not consider problematic can prove to be when occurring on a large scale in a population. For example, insurance fraud and small-scale tax evasion by millions of people costs the US government more than $320 billion dollars in revenue each year [10]. Such statistics beg the question of what so many different people could possibly have in common if dishonesty is indeed not innate. That is, if humans understand fairness on an intuitive level driven by both societal mechanisms and by social heuristics, what enables this high level cheating in specific situations?

There are many root causes of academic dishonesty, perhaps one of the most important of which is the idea of social conformity and societal pressure to succeed [11, 12]. Such societal factors have the ability to affect large masses of people, making them suitable candidates for explaining the widespread phenomenon of cheating. Many studies have shown that the people around us affect our behavior in subtle ways [13]. Part of the reason that cheating in high schools has reached such astronomical heights is that a self-perpetuating system of pressure and stress is running in America’s high schools. In an age of online statistics concerning just about everything, rising pressure is on students to succeed [14]. This increases stress, decreases actual performance, and increases cheating, part of a horrible cycle that has led some researchers to refer to cheating in high schools as an “epidemic “[5]. This issue is exacerbated by the fact that many teachers are not fully aware of the stress they may be causing their students [15]. The idea of necessary, objective success in school is a huge problem in our society, and has other implications such as depression, brought on by the dichotomous thinking promoted by many ill-advised parents. However, this is not the only thing that makes cheating as prevalent in American schools.

Conformity has been shown to have an incredibly powerful effect on people’s opinions and people's’ actions, sometimes even causing individuals to give an obviously incorrect answer just to fit in with a group that is blatantly wrong about something [16]. Despite this being a prime candidate for why immoral academic behavior is so prevalent in our schools, surprisingly little research has been done on the topic. Many students seem to (correctly) believe that many of their peers are cheating and use this to justify their actions [17, 18]. However, another study came up with a seemingly contradictory finding–cheating decreased significantly in classes that reported high student cohesiveness in their classroom [19]. Furthermore, that study also found that cheaters operated on less mature stages of moral development, thus suggesting that cheating was an individual choice and not a result of true vicarious learning. Since many of the few field studies done on the topic have been self-reported surveys, more research, particularly more direct observational research, needs to be done in order to find what the true correlation between various social interactions and cheating is. It is important to note that different types of social interactions (such as the ones between friends, family, or strangers) could lead to different results in terms of promoting or inhibiting dishonest behavior.

An important influence of cheating, and one that often goes unmentioned, is the effect of the environment on the rate of dishonest behavior. This is a difficult factor to study because most test-taking situations where cheating is directly observed by researchers are outside of the usual learning environment, such as a classroom. Also, surveys, the most common way to study dishonest behavior, cannot realistically expect the participant to recall an accurate answer about places where they cheated. Much of the research in this area has focused on asking students about their perceptions of the classroom and then surveying the participant about dishonesty there [19, 20]. The results of two different surveys conclusively found that different classroom settings have significantly different levels of cheating, with similar qualities describing classrooms with reduced levels of dishonesty in both the US and South Korea. This is important in and of itself because it shows that research about how social interaction and perception of the environment affects academic dishonesty in high school is probably generalizable to other societies. However, nearly all of the aforementioned studies fail to recognize the importance of the actual classroom in the dishonest experience. This oversight leads us to believe that some conclusions may not representative of academic cheating as it naturally occurs.

Previous studies have found that priming can make a significant difference on how much people falsify their scores; for example, thinking about the Ten Commandments reportedly eliminates statistically significant cheating. Some of those studies also found that creativity was an important factor in academic dishonesty, that is, more creative people cheated more. Interestingly, intelligence was not correlated with cheating in those studies[10, 21].

Different studies of cheating often come up with vastly different conclusions, probably due to the sheer number of variables that can impact dishonesty. For example, increasing the expected magnitude of reward for dishonesty was not found to significantly increase cheating. However, other studies have studied something similar and have come up with opposite or inconclusive results. One of the many variables that must be considered in such experiments is perceived punishment for dishonesty. The effect of punishment on cheaters was found to be dependent on the population, various environmental factors, and perceived benefit from dishonesty [22–24]. Gender is also an interesting factor to consider when studying academic dishonesty; many studies found that males cheat slightly more than females without a statistically significant result. For instance, a study of more than 270 students in Italy found no statistically significant difference between cheating in males and females, but proposed that a larger sample size or a different experiment might show one [25].

For this study, we collected and analyzed data from tests administered by teachers during class time in order to simulate the effect of the school environment on cheating. We delve into how various factors related to social behaviors relate to dishonesty, both individually, and when working in pairs.

## Materials and methods

All anonymized data is available online at https://sourceforge.net/projects/behavioral-correlates-cheating. The project was approved by the Massachusetts State Science and Engineering Fair SRC and the Lincoln-Sudbury Regional High School IRB. The project was reviewed and a form was signed (written consent) by three members of the school IRB. All participants gave written consent to the experiment, and consent on behalf of minors was given by participants’ parents. The IRB reviewed the method of consent used for minor participants in this study. Although participants were blinded to the goals during testing, teachers were instructed to debrief their students after all tests were administered.

In this study, we gave a matrix search task test to N = 243 students (N^individual^ = 161 split among 4 individual experimental conditions, and N^pairs^ = 82 split among 2 pair conditions) from different classes and grade levels at Lincoln-Sudbury Regional High School, a US high school located in Sudbury, MA. Participants were recruited through their teachers, who told students in their classes that this experiment would be taking place during class time. No exclusion criteria were applied: all students were allowed to participate, and participation was voluntary (see S1 Table for a table of descriptive statistics on participants).

The individual conditions consisted of a control and three experimental versions, which involved self-reported scores by the students and the recycling of other testing materials to eliminate any anxiety over repercussions. In particular:

- Control Condition: Students were told that their entire testing packet would be collected. At the end, the packets were collected and hand-graded (N = 42),
- Experimental Regular: before beginning the task, students were told that they would not be submitting the testing materials, and that test materials would be destroyed at the end of the session. We collected only a sheet with self-reported scores (N = 30).
- Experimental Friend (Priming): same as Experimental Regular, except that prior to the beginning of the task, participants were asked to list three qualities that described their best friend. (N = 60).
- Experimental Average (Priming): Same as Experimental Regular, except that prior to the beginning of the matrix search task, participants were told that an average number of matrices solved was 11 (this number was meant to be significantly higher than the expected average in the control). (N = 29).

**Fig 1:**
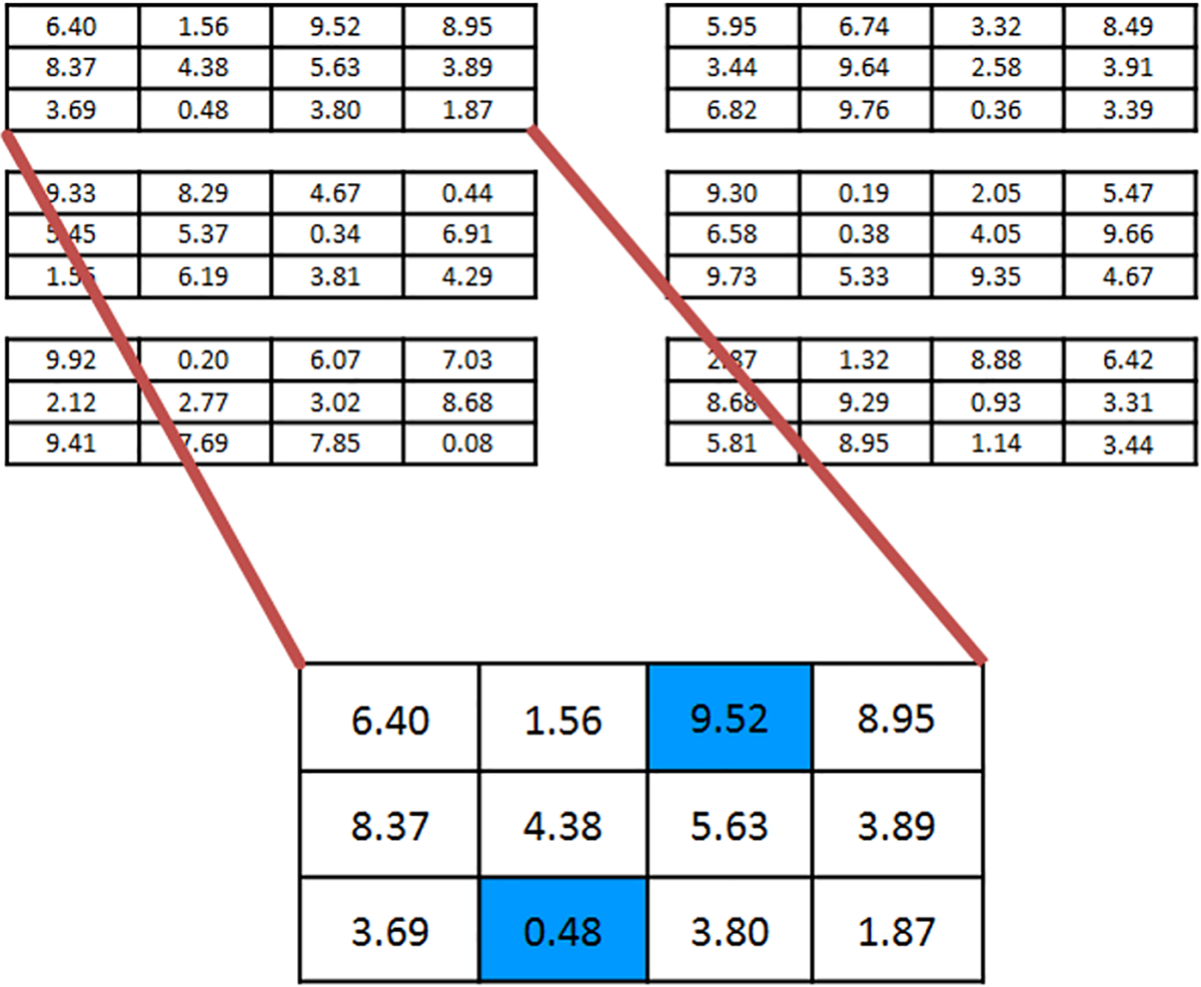
Schematic of portion of matrix search task test. The bottom of the figure shows a larger view of one of the matrices, with the correct answer filled in blue. The real task had 20 matrices (individual condition) and two independent sets of 20 matrices (for a total of 40) for the pair condition.

The pair versions were divided into a control (N = 21 pairs) and experimental (N = 20 pairs) section, with double the questions. For the pair experimental there was no priming, resulting in a test analogous to the plain self-reported version from the individual conditions. Pairs were assigned by a random number generator. We could see if people were dishonest by comparing the averages of the classes for the control and experimental groups–a large discrepancy would indicate cheating on the part of the experimental group. The test consisted of 20 or 40 (individual test and pair test, respectively) 4x3 matrices where the participant had to find the two numbers that sum up to 10 in as many matrices as they could in four minutes (see Fig 1 for schematic of task). In the instructions, students were told that four randomly chosen students from each class would receive a Jolly Rancher for every matrix they correctly solved.

After the test, participants were asked a series of post experimentation questions about how they perceived their honesty, intelligence, and popularity, as well as the class and the ‘block’ in which they were taking the test. We ranked the classes on a scale of 1–3, with 1 being the most difficult, to see if class difficulty correlated with score or the self-reported characteristics. The ‘block’ is a system of time used by the high school where we conducted this experiment, and was converted into a number 1–6 which represented when the class occurred during the day, with 1 being the earliest (at 7:50 A.M.). This data would be used to test if anything related to time, such as alertness, was correlated with academic dishonesty. We used peoples’ names to identify their gender. Outliers such as those with scores greater than the number of matrices given were removed from our analyses (N = 2). For this study, we mainly considered the individual conditions, although we also discuss some observations about the pair conditions as well.

## Results

**Fig 2:**
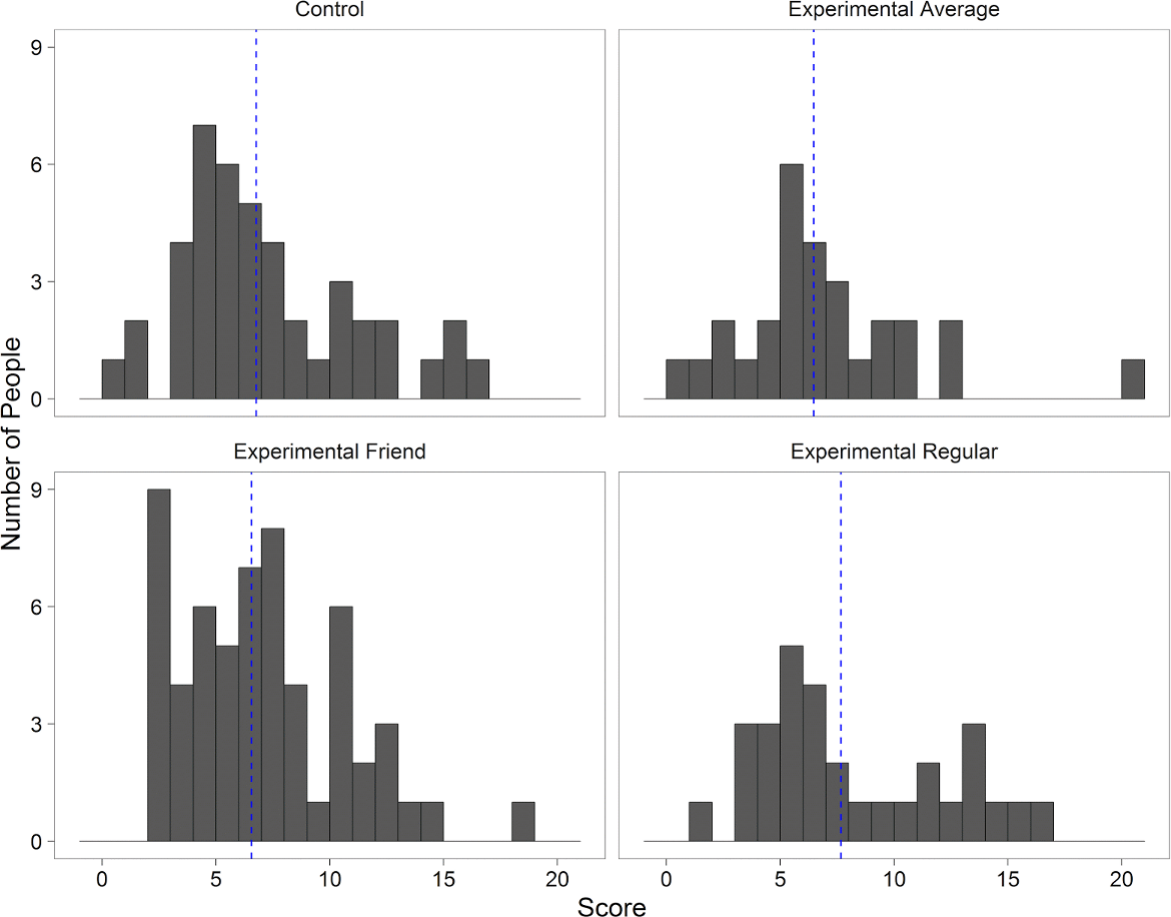
Histograms of scores in individual conditions. Blue line represents mean.

**Fig 3:**
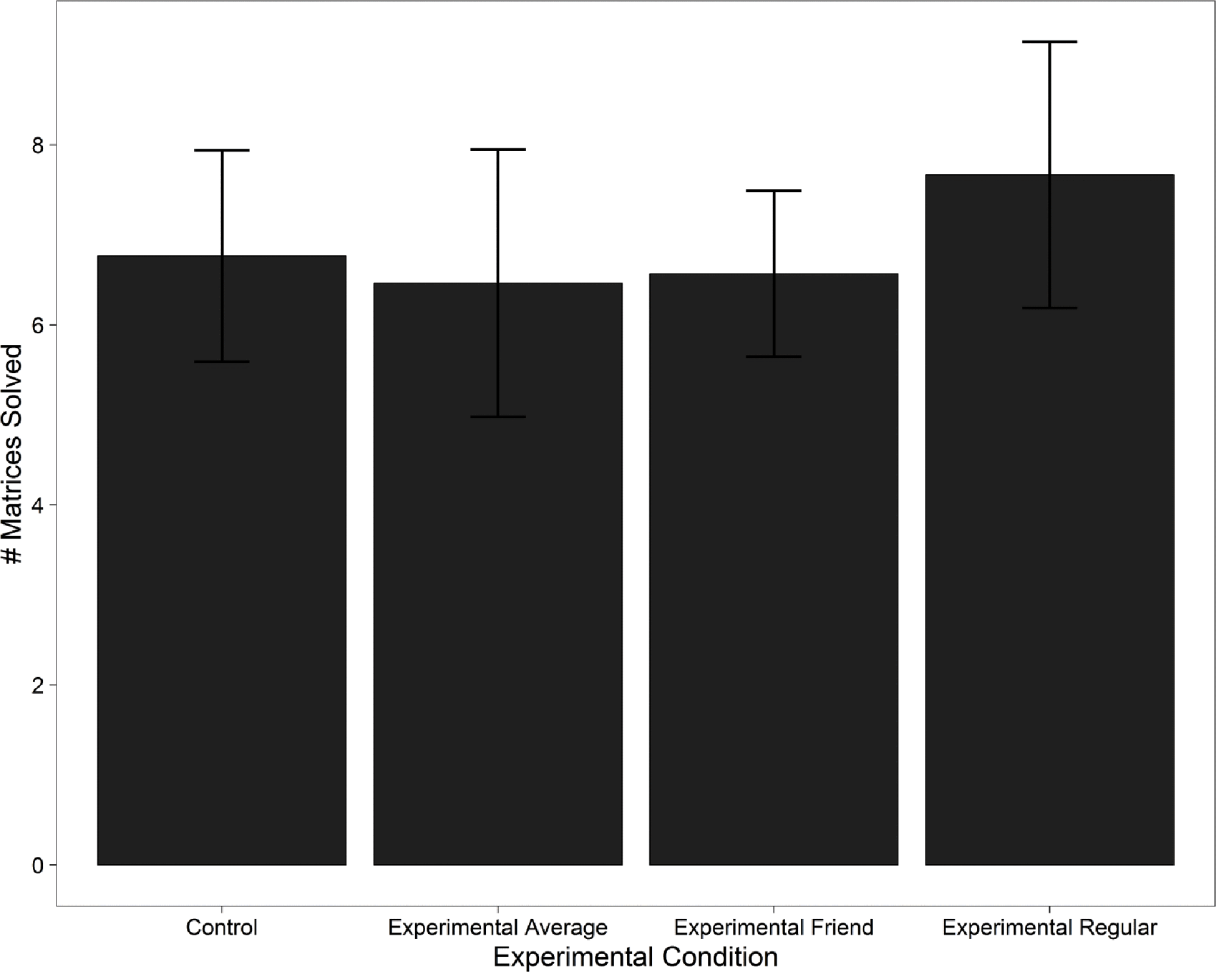
Mean number of matrices solved in the four individual experimental conditions. Error bars are 95% confidence intervals. While the distributions are visibly different between conditions (compare Fig 2), there is no significant difference in means between individual conditions.

Looking at the distribution of scores, the individual Control Condition is a positively-skewed unimodal distribution (median = 6, skewness = 0.67), while the Experimental Average group more closely resembles a normal distribution with an outlier (median = 6, skewness = 0.17 with the outlier removed). The large standard deviation of the scores of the control classes (SD = 3.93) is almost double that reported in prior literature using the same experimental task [10]. The Experimental Friend group is highly asymmetrical despite having the same median (median = 6), and is less spread out (SD = 3.27) than the Experimental Regular group (see Fig 2). Focusing on individual groups, we found no statistically significant difference between the means of the different designs (see Fig 3). A formal comparison of distributions with the Kolmogorov-Smirnov test was also not significant (p = 0.886), likely due to the sample size. However, the differing significance in regression models (such as dependence on gender in the experimental conditions but not in the control, which has been shown to be associated with cheating in the literature) leads us to believe that some cheating did occur. The lack of pronounced cheating may have been influenced by either the nature or the magnitude of the rewards. While the latter is in line with previous studies, the former may have had a significant effect due to the fact that the experiment took place in a classroom setting (see Discussion).

First, we examined how demographics (age, gender, number of siblings), self-perception (honesty, popularity, intelligence), and environmental factors (class level, block) influenced individual score across univariate models. Scores were not dependent on class level in either the control (p = 0.054) or an aggregate of the experimental conditions (p = 0.189), in accordance with literature that claimed that this task was not correlated with success in school (see S2 Table). Our study also found a difference between cheating in boys and girls. It was statistically significant for the Experimental Average condition (p = 0.032) with a large effect size of 3.4 extra matrices found among males, while the other experimental conditions were suggestive of a gender gap in performance, with boys performing better in all three. In the Control Condition, no such difference was present (p = 0.107), leading us to believe that boys cheated more than girls overall. Indeed, when we pooled all the experimental conditions together, the gender gap turned out to be statistically significant, with boys cheating more by about 1.8 matrices (p = 0.013; see S2 Table). We also found time of day to be negatively correlated with cheating: for each block later in the day, students in the experimental conditions ‘performed’ 0.5 matrices worse (p = 0.008). There was no such difference in the control (p = 0.545; see S2 Table), suggesting dishonesty in the self-reported scores. The additional variables were not significant predictors of score in univariate models (p > 0.05).

We also found interesting results concerning self-perception. In the Experimental Average condition, score and honesty were negatively correlated–a one point decrease in honesty corresponded to a score of an additional 1.2 matrices (p < 0.001; see S3 Table). Other demographic, self-perception, and environmental variables were ultimately not predictive of score. All of the aforementioned statistically significant results survived appropriate controls.

Interestingly, the absolute values of participants’ scores in our study were quite different from the literature. The mean score in our control group was 6.8 matrices answered correctly, more than 50% higher than were reported by similar studies [10]. Our standard deviation was also significantly larger than that shown in previous studies.

Potentially due to the sample size and to testing irregularities that emerged after the conclusion of the experiment in post-experiment interviews with teachers, none of the variables we tested for in the pair experiments were significant predictors of score. So, we will focus our analysis on the individual versions of the test. However, we note the interesting finding that tests done in pairs ‘underperformed’. That is, even in the control group, the mean did not even come near to having a mean double that of the individual tests (the pair average was just under 65 percent higher than the individual average).

## Discussion

This study strongly suggests that setting has a large impact on cheating behaviors. Some studies found in the literature were done in universities, but it appears as though they were not done in the classroom during regular class hours, all but negating the effect of the school environment on the study. While this is useful for certain things, since it eases restrictions on generalizing results, it does not accurately factor in the effects of societal norms on cheating. As we noted in the results, the distribution of scores in the control was very different from the experimental conditions, which leads us to believe that some people did in fact cheat, just not enough to make a noticeable difference in our relatively small sample size. Additionally, it is important to note that since participants were told in advance how they would be scored (self-reported or not), it is possible that those who knew they would be graded by an authority put in more effort than those who knew they could cheat. This might also contribute to the apparent lack of dishonesty in the experimental conditions. That is, lying may have at least partially compensated for the lack of effort.

This study further suggests that the type of reward is important in whether or not dishonesty will be widespread. The dampening of a cheating effect might mean that the classroom setting is prone only to one type of dishonesty, that is, cheating that concerns grades. If someone expects a very specific benefit from their actions, they may be less likely to cheat for a different reward of the same magnitude. This would make our rewards for success seem unimportant, and thus not result in the widespread cheating that is reported by surveys in high schools around the country. This idea makes sense because morality is enforced in schools outside of graded assignments, and seems to actually change the behavior of students for the better when things like grades are not concerned [26].

When all the conditions are pooled together, class level was found to correlate with score. This finding supports the notion that environment influences motivation as well–the data may suggest that students in more difficult classes were more motivated to do well even on something as unrelated to their studies as this matrix search task. This study confirms earlier findings that cheating is not correlated with intelligence, since different level classes had a similar difference between control and experimental conditions.

Self-reported honesty was found to be correlated with score in the Experimental Average condition, and although this result only appeared in one condition, we consider it important for several reasons. Firstly, it seems to support the idea that some cheating occurred, just not enough to be picked up by the Kolmogorov-Smirnov test. This is because the negative coefficient of the regression is in line with logic–higher scores, presumably achieved by cheating, result in lower self-reported honesty. Furthermore, this finding seems to stress the importance of the surroundings on participants–the presence of the average was the only difference between this and the Experimental Regular group, which leads us to believe that the very idea of norms brought on by the mention of an average made people self-report more truthfully than in other experimental groups.

Self-reported intelligence was correlated with score in the control, but not in the experimental conditions, leading us to believe that cheaters understand that their new score is not representative of their ability. The larger standard deviation in the experimental conditions, probably caused by sporadic dishonesty, made the correlation not statistically significant. We found that self-reported popularity was not significant in any case, possibly because of the open-ended nature of popularity. The negative correlation between time of day and dishonest behavior is interesting in that it suggests a possible link between alertness and cheating, and also because it could have important policy implications for testing. The small age range in the high school made correlation between it and score unlikely, and we accordingly found none.

We showed a statistically significant correlation between the score of males and the score of females, especially in the experimental conditions. An ANOVA on score that controlled for experimental condition and gender returned a statistically significant result (p = 0.005). Since there was no significant gender gap in the control, we believe that males cheated more when given the opportunity to do so. Furthermore, an overall model of all the individual versions also saw males performing better, likely due to dishonesty. Although for our study, age did not seem to be linked with this effect, the difference between this and an earlier study which found no gender gap in young children implies that genders begin to differentiate in morality sometime between 12 and 15. This makes sense in biological terms–more white matter in the brain develops the ability to cheat and lie. Also, adolescent boys are known for being significantly more impulsive than their female counterparts, which might cause them to cheat once and get trapped in the endless cycle of cheating and stress. This result is especially intriguing because it shows that maturity, which generally increases with age, does not consistently offset the biological and environmental factors that make cheating more likely.

As mentioned earlier, pairs significantly underperformed when compared to individuals taking the test. This is probably due to two factors: any cheating that occurred in the individual versions was negated because of limited time to cooperate, and time constraints did not allow for proper divvying up of work and focus on the completion of the task. Thus, teachers might use group work on certain tasks to reduce widespread cheating. However, they would have to remember that two people in a group work at a different pace than as individuals, a fact that has been often overlooked in the literature.

## Conclusion

We generated a matrix search task test which we distributed to 16 high school classes (N = 243) in the form of six different experimental conditions. The participants were then asked the time they were taking the test, their age, their perceived intelligence, popularity, and honesty. We ranked the classes they were taking the test in by level of difficulty. The most important findings were: (1) the societal norms that go hand in hand with test-taking in school as administered by a teacher significantly dampen small-scale cheating–perhaps suggesting a trend of environment-specific rewards setting off cheating; (2) reminding participants about societal norms by giving them an average score made people report honesty more truthfully; (3) a matrix search task is appropriate for these kind of studies because results do not correlate with scholastic achievement; (4) males seem to cheat more than females; and (5) later time of day dampens cheating in high school students. Dishonesty in school has important implications in the lives of students after high school or college, and the various factors that influence and maintain the prevalence of cheating in America’s public school systems are critical to curbing the massive damage caused by seemingly minor dishonesty on the parts of millions of people in things like tax returns and insurance claims. Furthermore, the idea that situational rewards seem to affect cheating rates is interesting and could be important in curbing academic dishonesty in the future.

For future work, it would be interesting to examine the idea of environment-dependent rewards, which have been suggested by a few studies in the literature, much further. This would involve looking at different settings, which would likely give vastly different results. For instance, a child might be willing to act in a dishonest fashion to obtain a balloon at a party, but be less inclined to cheat on a similar task when alone.

It would be interesting to look at the responses to the priming question to see how certain words correlate with score. For instance, are people who answer “honesty” less likely to cheat? This might require a larger sample size and additional data collection. Further research might also concern the gender gap between dishonesty, and what factors influence how wide it is. We could also examine different types of cheating, such as copying or plagiarism, and how the results differ from the ones presented here. This research also raises the question of how participants would fare on untimed or longer tests, especially in the pair groups. Further investigation into cheating in different types of classes might yield interesting results: for example, might people in English classes cheat more than those in math classes?

Examining different age ranges for this type of behavior might help solidify a link between adolescence and cheating, and perhaps showing the ‘tipping point’ (if it exists) where maturity starts to override impulse in cases of academic dishonesty. A larger sample size could allow for further analysis of how tiredness and alertness affect dishonesty. Studies could also be done to examine if a person cheats more if a situation is perceived to be unfair or a task is seemingly undoable, which could involve putting unreasonable time constraints on a task or giving another (fake) participant an obvious advantage. Groups of more than two could also be examined, as could the effect of doing a group activity first and then taking the test individually. Dishonest behavior in various forms pervades everyday life, and we expect that studying not only the behavior but also the context in which it occurs will lead to a better understanding of how to mitigate it.

### Ethics statement

IRB permission was obtained prior to conducting experiments. Participants all signed informed consent forms,and consent on behalf of minors was given by participants’ parents. The IRB reviewed the method of consent used for minor participants in this study. Data was anonymized to preserve confidentiality.

## Supporting information

S1 Table Descriptive characteristics of participants. ^1^Gender reported as a percentage.
(DOCX)

S2 Table Regression of Score on Class Level. Univariate regressions with tobit model of Score on Class Level, Gender, and Block for the control condition (left) and all experimental conditions (right).
(DOCX)

S3 Table Regression of Score on honesty. Regression with tobit model of Score on self-reported Honesty for the control condition (left) and the experimental condition with the average given (right).
(DOCX)

S4 Table Regression of Score on Demographics, Self-Perception, and Environmental Factors. Multivariate regression with tobit model of Score on Demographics, Self-Perception, and Environmental Factors for the control condition (left) and all experimental conditions (right).
(DOCX)

S5 Table Regression of Score on experimental condition. Regression with tobit model of score on treatment dummies.
(DOCX)
