# Supplementary material for: Behavioral correlates of cheating: Environmental specificity and reward expectation
Source: PLoS One. 2017 Oct 26;12(10):e0186054. doi: 10.1371/journal.pone.0186054 (PMC5657619; doi:10.1371/journal.pone.0186054)
Supplement: S1 Table — 1Gender reported as a percentage. (DOCX) [file pone.0186054.s001.docx]

|  | Mean | Standard Deviation | Range |
| --- | --- | --- | --- |
| Age | 16.78 | 1.15 | [14, 19] |
| Self-reported intelligence | 7.15 | 1.49 | [1, 10] |
| Self-reported honesty | 7.70 | 1.82 | [0, 10] |
| Self-reported popularity | 6.06 | 1.91 | [0, 10] |
| Block | 3.42 | 1.79 | [1, 6] |
| Gender (% Male) | 45.74^1^ |  |  |
