# Supplementary material for: Behavioral correlates of cheating: Environmental specificity and reward expectation
Source: PLoS One. 2017 Oct 26;12(10):e0186054. doi: 10.1371/journal.pone.0186054 (PMC5657619; doi:10.1371/journal.pone.0186054)
Supplement: S2 Table — Univariate regressions with tobit model of Score on Class Level, Gender, and Block for the control condition (left) and all experimental conditions (right). (DOCX) [file pone.0186054.s002.docx]

|  | *Dependent Variable:*  *Score (Control)* | | | *Dependent Variable:*  *Score (Experimental)* | | |
| --- | --- | --- | --- | --- | --- | --- |
|  | *Coef.* | *S.E.* | *P* | *Coef.* | *S.E.* | *P* |
| Class Level | -1.288 | 0.670 | 0.054 | -1.084 | 0.8260 | 0.189 |
| Gender (Male) | 1.931 | 1.198 | 0.107 | **1.781** | **0.713** | **0.013** |
| Block | -0.271 | 0.448 | 0.545 | **-0.511** | **0.193** | **0.008** |
| N |  | 42 |  |  | 125 |  |
