# Supplementary material for: Behavioral correlates of cheating: Environmental specificity and reward expectation
Source: PLoS One. 2017 Oct 26;12(10):e0186054. doi: 10.1371/journal.pone.0186054 (PMC5657619; doi:10.1371/journal.pone.0186054)
Supplement: S3 Table — Regression with tobit model of Score on self-reported Honesty for the control condition (left) and the experimental condition with the average given (right). (DOCX) [file pone.0186054.s003.docx]

|  | *Dependent Variable:*  *Score (Control)* | | | *Dependent Variable:*  *Score (Experimental w/ Average)* | | |
| --- | --- | --- | --- | --- | --- | --- |
|  | *Coef.* | *S.E.* | *P* | *Coef.* | *S.E.* | *P* |
| Honesty | -0.498 | 0.328 | 0.129 | **-1.244** | **0.251** | **0.000** |
| N |  | 42 |  |  | 29 |  |
