# Supplementary material for: Behavioral correlates of cheating: Environmental specificity and reward expectation
Source: PLoS One. 2017 Oct 26;12(10):e0186054. doi: 10.1371/journal.pone.0186054 (PMC5657619; doi:10.1371/journal.pone.0186054)
Supplement: S4 Table — Multivariate regression with tobit model of Score on Demographics, Self-Perception, and Environmental Factors for the control condition (left) and all experimental conditions (right). (DOCX) [file pone.0186054.s004.docx]

|  | *Dependent Variable:*  *Score (Control)* | | | *Dependent Variable:*  *Score (Experimental)* | | |
| --- | --- | --- | --- | --- | --- | --- |
|  | *Coef.* | *S.E.* | *P* | *Coef.* | *S.E.* | *P* |
| Block | -0.769 | 0.479 | 0.109 | **-0.524** | **0.180** | **0.004** |
| Honesty | **-0.702** | **0.341** | **0.040** | **-0.514** | **0.178** | **0.004** |
| Gender (Male) | 1.975 | 1.128 | 0.080 | 1.038 | 0.682 | 0.128 |
| N |  | 42 |  |  | 125 |  |
