# Supplementary material for: Behavioral correlates of cheating: Environmental specificity and reward expectation
Source: PLoS One. 2017 Oct 26;12(10):e0186054. doi: 10.1371/journal.pone.0186054 (PMC5657619; doi:10.1371/journal.pone.0186054)
Supplement: S5 Table — Regression with tobit model of score on treatment dummies. (DOCX) [file pone.0186054.s005.docx]

|  | *Dependent Variable:*  *Score (All Conditions)* | | |
| --- | --- | --- | --- |
|  | *Coef.* | *S.E.* | *P* |
| Experimental Condition w/ Average Given | -0.289 | 0.945 | 0.759 |
| Experimental Condition w/ Friend Priming | -0.162 | 0.783 | 0.836 |
| Experimental Condition w/o Friend Priming | 0.936 | 0.924 | 0.311 |
| Block | **-0.637** | **0.205** | **0.002** |
| Honesty | **-0.534** | **0.154** | **0.000** |
| Gender (Male) | **1.384** | **0.586** | **0.018** |
| N |  | 119 |  |
